# Wavefront Characteristics of a Digital Holographic Optical Element

**DOI:** 10.3390/mi14061229

**Published:** 2023-06-10

**Authors:** Beom-Ryeol Lee, José Gil Marichal-Hernández, José Manuel Rodríguez-Ramos, Wook-Ho Son, Sunghee Hong, Jung-Young Son

**Affiliations:** 1 CG/Vision Section, Electronics and Telecommunications Research Institute, Daejeon 34129, Republic of Korea; lbr@etri.re.kr (B.-R.L.); whson@etri.re.kr (W.-H.S.); 2 Industrial Engineering Department, Universidad de La Laguna, 38200 La Laguna, Spain; jmariher@ull.edu.es (J.G.M.-H.); jmramos@wooptix.com (J.M.R.-R.); 3 Research & Development Department, Wooptix S.L., 38204 La Laguna, Spain; 4 Hologram Research Center, Korea Electronics Technology Institute, Seoul 03924, Republic of Korea; shhong@keti.re.kr; 5 Public Safety Research Center, Konyang University, Nonsan 32992, Republic of Korea

**Keywords:** digital HOE, optical performance, phase map, reconstructed wavefront, Shack–Hartmann sensor, Zernike polynomials

## Abstract

In this study, a 50 × 50 mm holographic optical element (HOE) with the property of a spherical mirror was recorded digitally on a silver halide photoplate using a wavefront printing method. It consisted of 51 × 96 hologram spots with each spot measuring 0.98 × 0.52 mm. The wavefronts and optical performance of the HOE were compared with those of reconstructed images from a point hologram displayed on DMDs of different pixel structures. The same comparison was also performed with an analog-type HOE for a heads-up display and with a spherical mirror. A Shack–Hartmann wavefront sensor was used to measure the wavefronts of the diffracted beams from the digital HOE and the holograms as well as the reflected beam from the analog HOE and the mirror when a collimated beam was incident on them. These comparisons revealed that the digital HOE could perform as a spherical mirror, but they also revealed astigmatism—as in the reconstructed images from the holograms on DMDs—and that its focusability was worse than that of the analog HOE and the spherical mirror. A phase map, i.e., the polar coordinate-type presentation of the wavefront, could visualize the wavefront distortions more clearly than the reconstructed wavefronts obtained using Zernike polynomials. The phase map revealed that the wavefront of the digital HOE was more distorted than those of the analog HOE and the spherical mirror.

## 1. Introduction

A hologram is a three-dimensional photograph of an object that is recorded by a coherent laser beam [1]. It consists of interference fringes formed by the interference between the reference and object beams, which stem from the laser beam. The digital recording of holograms started with the Zebra hologram [2]. The Zebra hologram is a three-dimensional photograph generated from a two-dimensional array of hologram spots. Each hologram spot in the array is the hologram made from an object that is a view image in a multiview image set that has the same array dimensions as the hologram spot array. This Zebra hologram recording method was utilized to record a microlens array on a photopolymer film [3] by replacing each view image with the computer-generated hologram (CGH) [4,5] of a lens or spherical mirror. This method was further developed as a wavefront printing method for recording digital holographic optical elements (HOEs) [3]. The traditional method for creating an HOE with an optical component is recording by interfering with either the rays reflected from or transmitted through the component itself or the object beam with the laser beam incident to the component as the reference beam. This is the typical method for recording analog-type HOEs [6]. However, this recording method has the serious problem of requiring optical components of various sizes and focal lengths to record HOEs with the desired focal lengths and sizes. As the desired HOE size increases, recording becomes more difficult because it requires a larger component with the desired focal length, a higher-power laser beam, and a larger space in which to install the recording setup. The wavefront recording method is intended to overcome this problem. It holographically records the hologram fringe pattern of the optical component instead of the rays from the object itself on a holographic photoplate or photographic film. Using this method, the fringe pattern is generated by a computer, i.e., a computer-generated hologram (CGH), and this is segmented equally into a desired number of pieces. Each piece in the segmented CGH is focused with a desired spot size equal to that of the beam from the object on the plate or film and is recorded as a hologram spot using a collimated reference beam that is directly incident to the plate/film with a designed incident angle. For this purpose, each fringe piece is displayed on a spatial light modulator (SLM) [7] and projected through focusing optics onto the plate/film, which is also virtually segmented into the same number of segments as the fringe pieces. In this way, the relative position of the fringe piece in the segmented CGH becomes the same as that of its hologram spot in the plate or film. Hence, the plate/film consists of a hologram spot array. When a reconstructed beam illuminates the plate/film, each hologram spot will reconstruct its fringe piece, and the complete fringe pattern will be reconstructed to work as the optical component represented by the fringe pattern. Hence, the plate/film becomes a digital HOE. In theory, this digital HOE has no size limit if the available photoplate or photographic film is the same size as the desired HOE because the CGH can be made to have any size. Because the CGH of the optical components, such as the lens and the spherical mirror, has the same fringe pattern as the Fresnel zone plate [8], the zone plate can be used instead of the hologram fringe pattern.

The usability of the digital HOE as an optical component depends solely on its optical performance, which can be measured in two ways: one is by measuring the focusing characteristics of the HOE with a collimated beam, and the other is by measuring the wavefront using a Shack–Hartmann wavefront sensor [9,10]. This sensor can provide information relating to the wavefront aberration introduced into the input beam by the object through which the beam passes. The information is obtained by calculating the centroid deviation of each light spot in a light spot array that is formed by a microlens array when the beam passes through it [11]. The deviation can be used to draw a phase map to visualize the wavefront aberration, to show the converging characteristic of the reflected/transmitted beam from the object, and to calculate various optical aberrations in the object with the use of Zernike polynomials [12,13]. The polynomials have been used to plot the refraction error in an optical component [14]. The sum of all these aberrations reconstructs the wavefront of the object. Because the technology used to create digital HOEs based on the wavefront printing method is not yet mature, its optical performance is compared with that of a spherical mirror, an analog HOE for a heads-up display [15], and reconstructed images of point holograms that are displayed on DMDs of two different types, i.e., rhomb- and square-pixel types. The DMD is a reflection-type spatial light modulator (SLM) with a frame speed of more than 100,000 frames/s [16]. In displaying holograms, it has the advantage of enabling the reconstructed images to be separated from the reconstruction beam even for on-axis holograms because of its pixel rotation of either 12° or −12°. This makes the DMD a binary display chip. The fringe pattern of the hologram displayed in the DMD does not differ from that recorded in the digital HOE except for the details of the pattern—each hologram spot in the digital HOE has a size of 0.98 × 0.52 mm, and there are 4096 × 2160 SLM pixels within each spot, whereas the DMD exhibits 1280 × 800 pixels within 10 × 6 mm. This means that the resolution of the digital HOE pattern is more than 1200 times higher than that of the DMD. However, the main difference between them is in their image reconstruction processes. In the case of holograms on DMD, the fringe pattern forms the phase conjugated beam when it is illuminated by a reconstruction beam. Hence, the pattern is converged to a focused spot to form the reconstructed image of an object point, i.e., it works in the same way as a lens or spherical mirror. In the case of digital HOEs, each segmented piece of the pattern is reconstructed using its corresponding hologram spot and then combined with other reconstructed segmented pieces to form the full fringe pattern. This pattern works as either a lens or a spherical mirror to enable the reconstruction beam to be focused as a light spot. Hence, both the fringe pattern on the DMD, and analog and digital HOEs, work as a lens or a spherical mirror.

In this study, the wavefront aberrations and focusing characteristics of digital HOEs with spherical mirror properties were measured using a Shack–Hartmann wavefront sensor. The same measurements were performed for a spherical mirror, an analog HOE, and reconstructed images from a point hologram displayed on both rhomb- and square-pixel type DMDs. In order to compare their focusing and wavefront aberration characteristics with those of the digital HOEs, a wavefront deviation map, a phase map, and reconstructed wavefronts from Zernike polynomials were calculated.

## 2. Setups for Recording and Measuring the Optical Characteristics of Digital HOEs

**Figure 1:**
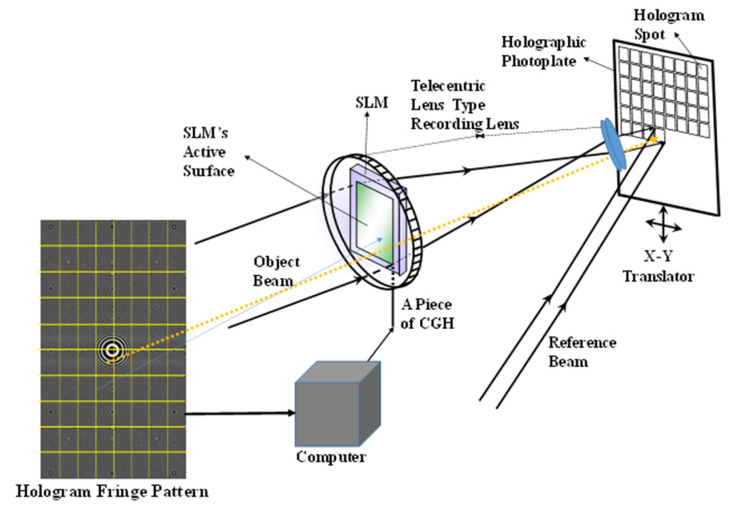
Digital HOE recording setup: each segment of the hologram fringe pattern generated using a computer is recorded as a hologram point on its corresponding location on the photoplate.

As explained above, the optical setup for recording a digital HOE is shown in Figure 1.

**Figure 2:**
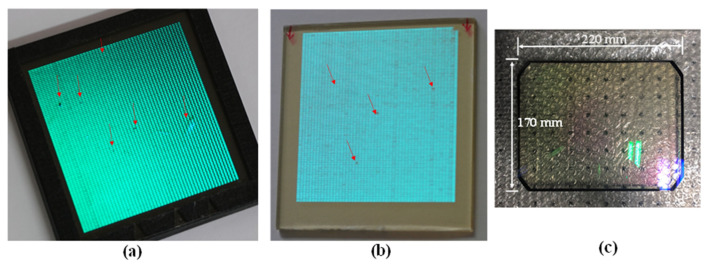
The two digital and analog HOE samples. Arrows in the samples indicate defective hologram points. These were mainly underdeveloped: (**a**) sample 1, (**b**) sample 2, and (**c**) analog HOE.

The setup is the same as that used to record Zebra holograms. As the object beam, a collimated laser beam illuminates the lens in front of an SLM and then passes through the active surface of the SLM, which displays a piece of the hologram fringe pattern. The wavefront of the beam is modulated by the pattern and is further converged to a point at the focal point of the lens in front of the photoplate. The beam that passes through the lens is collimated to the size of the hologram spot and is incident normally to the photoplate. This beam interferes with the reference beam, which is incident to the photoplate at a designed angle, on the surface of the photoplate. The two lenses in the setup work in the same way as a telecentric lens because the collimated beam from the second lens maintains a constant size for all the hologram spots on the photoplate. With the exception of the photoplate, the optical setup is fixed to an optical table. The photoplate is on the X–Y translation stage, which is fixed to the same optical table. The stage translates the photoplate to both the X- and Y-axis directions corresponding to the size of the hologram spot. As shown in Figure 1, if the hologram fringe pattern is segmented into 8 (horizontal) × 10 (vertical) pieces of equal size, the photoplate is also virtually divided into the same number of equally sized areas as the pattern, as specified in the rectangular array. Then, from the first piece on the left of the top line, it is displayed on the active surface of the SLM and recorded as a hologram spot on the area furthest to the left of the top line of the photoplate. Next, the second piece will be displayed on the SLM and recorded on the area next to the first hologram spot, and the third piece on the area next to the second hologram spot, and so on until the eighth piece is recorded. After recording the pieces on the first line, the first piece on the left of the second line from the top is recorded on the second line from the top of the photoplate. The recording continues with the same sequence as the first line pieces until the pieces for the last line are recorded. The rectangular array on the photoplate represents the recorded hologram spots and the broken line indicates the piece of the fringe pattern and its corresponding hologram spot that will be recorded on the photoplate. However, the digital HOE used in this study was recorded using an optical setup that was slightly different from Figure 1 because a reflection-type SLM was used instead of the transmission-type SLM shown in Figure 1. The HOE was recorded on a 50 × 50 mm silver halide photoplate. This was mounted on the X–Y translator and translated to 0.98 mm in the horizontal direction for each hologram spot in the same line and to 0.52 mm in the vertical direction for the spots in the next line. This translation allowed 51 × 96 spots to be recorded. Each spot measured 0.98 × 0.52 mm. The SLM used was a SONY LCoS (Liquid crystal on Silicon) with a resolution of 4096 × 2160 and a pixel size of 3.74 × 3.74 μm [17]. This meant that the LCoS SLM had an active surface area of 16.33 × 8.67 mm. The object beam with a wavelength of 532 nm, which was collimated to have a diameter of more than 16.33 mm, illuminated the active surface of the SLM. The beam reflected from the surface was collimated using the telecentric lens to have a rectangular shape of 0.98 × 0.52 mm and was normally incident to the photoplate. The reference laser beam with the same wavelength as the object beam was incident to the rectangle with a 38° beam incident angle. Hence, each box in the photoplate would be filled with a hologram spot measuring 0.98 × 0.52 mm. When this hologram spot was reconstructed, the reconstructed fringe piece measured 0.98 × 0.52 mm. To reconstruct the full fringe pattern, this fringe piece was combined with those from other hologram spots with a minimum gap or overlap between them. Figure 2 shows the two HOE samples recorded on the 50 × 50 mm silver halide photoplate. These are referred to as samples 1 and 2. The contrast of the hologram spots in sample 2 were slightly worse than those of sample 1 because of the short development time; the hologram spots do not have a rectangular shape and are smaller in size than those of the rectangle. The smaller size made the gaps between the spots wider. The gap size was almost 1/3 of the 0.52 mm, i.e., approximately 0.17 mm. Because of their small size, the positions of the spots within the rectangular area also varied. Some defective hologram spots were also visible, as indicated by the arrows. Figure 2 also shows an analog HOE measuring 220 × 170 × 16 mm (thickness). This HOE was just a spherical mirror with a focal length of 500 mm. A green reflective coating was applied to the mirror’s surface. The surface was covered by a plano-convex lens with the same radius of curvature as the spherical mirror. Therefore, the HOE was acting as the spherical mirror for the spectral range covered by the green coating. For the other visible spectral range, it was transparent to operate as a heads-up display screen.

**Figure 3:**
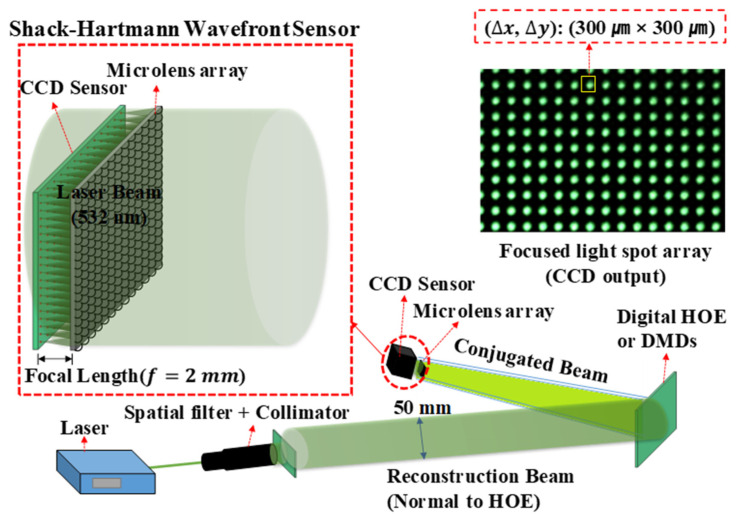
Optical setup for measuring the wavefront of the diffracted beam from the digital HOE. The focused light spot array was introduced using a Shack–Hartmann wavefront sensor, which was located close to the focal area of the diffracted beam, i.e., the phase conjugated beam.

The optical characteristics of the HOE were measured using the optical setup shown in Figure 3. This consisted of a laser with a wavelength of 532 nm and laser beam collimation optics, digital HOE/holograms on DMDs, and a Shack–Hartmann wavefront sensor. The collimated laser beam with a diameter of 50 mm was incident to the HOE with a 38° beam incident angle in order to illuminate the entire surface of the HOE and the reflected beam from the surface, which was normal to the HOE surface and propagating to the wavefront sensor. In the case of the hologram, the collimated beam was normally incident to the active surfaces of the DMDs and the phase conjugated beam propagated along the beam incident plane of the collimated beam with ±24° separation from the collimated beam, to the wavefront sensor. The sensor consisted of a microlens array with 77 × 51 lenses and a CCD detector with a resolution of 6000 × 4000. Each microlens in the array had a square shape with a side length 300 μm and the pixel size of the detector was 3.89 μm × 3.89 μm. This gave the detector a size of 23.34 × 15.56 mm and each microlens had 77 × 77 (300/3.89) pixels. Because the focal length of each microlens in the array was 2 mm, the detector surface and the microlens array were positioned parallel to each other 2 mm apart. The 77 × 77 pixels allowed the wavefront aberration of ±4.28° in both the horizontal and vertical directions to be detected. Because the sensor was installed in a SONY mirrorless camera without a camera lens, it was easy to replace the sensor with another SONY mirrorless camera for the measurement of the focusing characteristics. When a beam was incident to the sensor, the detector output would be a light spot array, as shown in Figure 3. Each microlens in the array formed one of the light spots on its “hired” 77 × 77 pixel arrays. The position of a spot in the pixel array can be represented by a quadrant value when the center of the array is considered as the origin. So, the maximum value of the spot position is ((±)38.5, (±)38.5). The wavefront aberration is represented by the quadrant values of the spot position. Hence, the wavefront aberration can be visualized by simply plotting the spot position with the pixel number unit and/or by the square root value of the position. The latter is similar to a polar coordinate representation of the spot positions and different colors can be assigned to the different value ranges of the spot positions. This produces a color phase map that visualizes the wavefront. For the phase map, the square root values of the spot positions are sorted with a 2.5-pixel distance interval. Then, white, blue, green, red, cyan, magenta, yellow, and so on, are, respectively, assigned to pixel distance deviations of 0 to 2.5, 2.5 to 5.0, 5.0 to 7.5, 7.5 to 10.0, 10.0 to 12.5, 12.5 to 15.0, 15.0 to 17.5, 17.5 to 20.0, and so on. When the 38.5 pixels are divided by 2.5 pixels, 16 different colors are needed, but when the diagonals are considered, $\left(\sqrt{2}\times 38.5\right)/2.5≅22$ colors are required. To reduce the required number of colors, the pixel interval can be changed to give nλ (where n=1,2,3,⋯,11, and λ is the wavelength of the laser beam) path length differences from the focal length of each microlens. Table 1 summarizes the wavefront aberration angles corresponding to nλ (Table 1a) and 2.5 m (Table 1b), where *n* and m are 1 to 11 and 1 to 16, respectively. This color phase map allows the wavefronts and the focusability of the HOE and the spherical mirror to be visualized. The total number of spots was 69 × 43 = 2967 because some of the microlenses at the edges of the microlens array were damaged in the process of installing the array on the detector of the mirrorless camera. Four spots were removed from each side of the array, i.e., eight spots for both the vertical and horizontal sides were removed from 77 × 51 spots.

The performance of the wavefront sensor was first tested using the reconstructed images of the point hologram, which was displayed on the two different types of DMD, i.e., rhomb- and square-pixel type DMDs. Because each pixel of the rhomb-pixel DMD was rotating along its vertical diagonal and those of the square-pixel DMD either along their 45° diagonal or −45° diagonal, it was expected that the wavefront aberration would appear differently.

## 3. Focusing and Wavefront Characteristics of Reconstructed Images from DMDs

**Figure 4:**
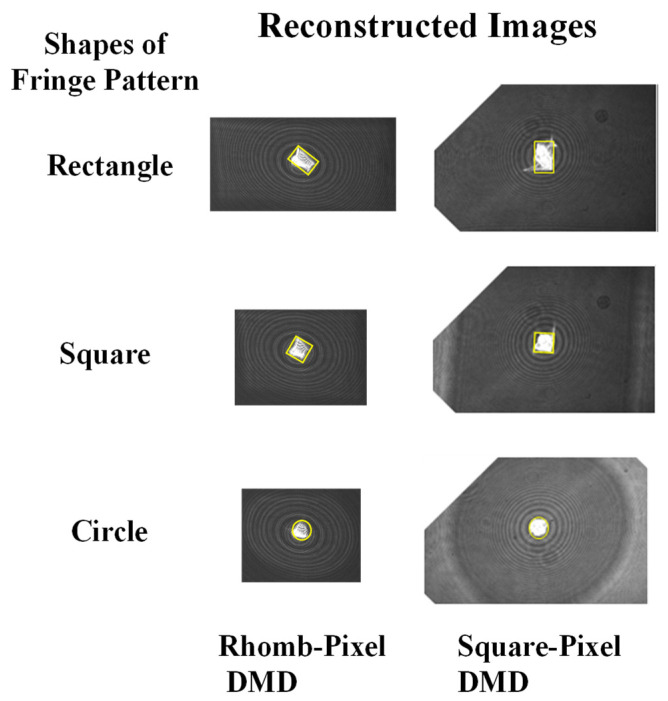
Reconstructed images from the holograms displayed on both rhomb and square. The image shape is the same as the hologram shape displayed on the DMDs.

**Figure 5:**
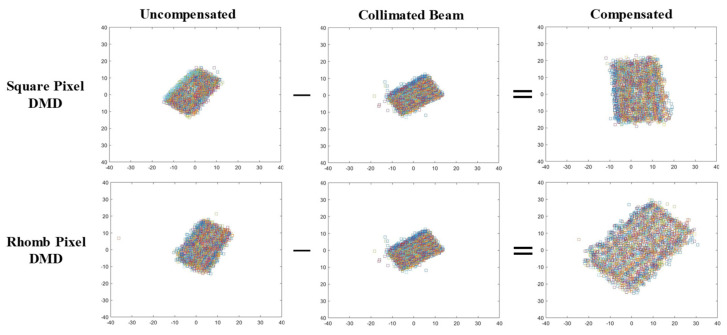
Wavefront deviation distributions obtained by plotting the quadrant value of each of the 69 × 43 spots. The compensated distribution means that they are rotated 45° compared with the rotation axis of their corresponding DMDs.

Because the smallest pixel size of currently available SLMs is 3.74 μm, as mentioned above, the maximum crossing angle between the reference and object beams cannot exceed 4.076° for a hologram recording. This angle means that the distance between the object and reference beams should not exceed 35.6 mm at a 500 mm distance from the hologram. As explained above, because the active surface size of the SLM is 16.33 × 8.67 mm, off-axis holograms can be recorded if the object is at a distance of more than 223 mm. However, because most SLMs have pixel sizes greater than the 3.74 μm, an off-axis hologram cannot be recorded unless the object distance is increased. The DMDs used in this paper were a rhomb-pixel DMD with a pixel resolution of 1280 × 800 and a pixel size of 7.637 μm × 7.637 μm, and a square-pixel DMD with a pixel resolution of 1920 × 1080 and a pixel size of 10.8 μm × 10.8 μm. These pixel sizes meant that the crossing angle between the object and reference beams could not exceed 1.996° and 1.411° for the rhomb and the square, respectively. The crossing angles meant that the distances between the object beam and the reference beam could not exceed 17.42 mm and 12.3 mm for the rhomb and the square, respectively, at a distance of 500 mm. Because the dimensions of the rhomb and the square were 10 × 6 mm and 20 × 10 mm, respectively, an off-axis hologram could not be recorded on the square-pixel DMD. Therefore, on-axis point holograms were prepared for both DMDs for the comparison. Figure 4 shows the reconstructed image of the point. Because each DMD chip was rectangular, the points had three hologram fringe patterns: a rectangle filling the entire active surface area, squares, and a circle fitting to the smaller side dimension of each chip. The image had the same shape as its corresponding hologram pattern, though it was rotated to 135° for the rhomb and 90° for the square. This image shape indicated that the pattern itself formed the phase conjugated beam. The image sizes of the rhomb and the square for the rectangular shape fringe patterns were 500 × 600 μm and 1.3 × 1.0 mm, respectively. The image size of the square was approximately four (1.3/0.3) times of that of the rhomb. Figure 5 shows the wavefront deviation distributions obtained using the wavefront sensor from the reconstructed images of the holograms on both the rhomb- and square-pixel DMDs shown in Figure 4. Figure 5 shows the wavefront deviation distribution obtained by plotting the quadrant value of each of the 69 × 43 spots. The scale of both the horizontal and vertical axes was ±40 pixels. The deviations were distributed in the form of rectangles but were rotated 48° and 58° for the square-pixel and the rhomb-pixel DMDs, respectively. The rectangular forms originated from the shape of the microlens array, but they also indicated that the deviations increased when the microlens was further away from the center of the microlens array. The distributions were extended from −12 to 16 pixels in the vertical direction and −15 to 12 pixels in the horizontal direction for the square, and from −14 to 18 pixels in the vertical direction and −10 to 16 pixels in the horizontal direction for the rhomb. The deviation distribution of the rhomb was slightly wider than that of the square. This resulted from the slightly higher converging angle of the phase conjugated beam of the rhomb compared with that of the square. As described above, the image size of the square was more than four times that of the rhombus, but its active area was slightly less than four times that of the rhomb. This meant that the converging angle of the square was slightly smaller than that of the rhomb. The aberrations of both DMDs were slightly shifted upwards but slightly shifted to the left for the square and to the right for the rhomb. These wavefront deviations should be compensated by that of the collimated beam because it was difficult to obtain an ideally collimated beam with no wavefront deviation, i.e., 0 deviation. The wavefront aberration of the collimated beam was added to those of the reconstructed images. The wavefront deviation distribution of the collimated beam had the same rectangular shape but was rotated by 26°. The deviation was extended from −12 to 14 pixels in the horizontal direction and from −12 to 12 pixels in the vertical direction. This aberration was almost symmetrically distributed and smaller than those of the reconstructed images. When this aberration was extracted from those of the reconstructed images, the deviation distributions of the reconstructed images took the form of a 92° rotated rectangle for the square and a 45° rotated rectangle for the rhomb. These rotation angles closely matched the rotated angle of their corresponding reconstructed images, although the angle was 45° instead of 135° for the rhomb. They were in symmetry. This might be caused by the wavefront inversion by each microlens in the wavefront sensor microlens array. The deviations are distributed from −16 to 20 pixels in the vertical direction and −9 to 15 pixels in the horizontal direction for the square, and from −25 to 28 pixels in the vertical direction and −20 to 28 pixels in the horizontal direction for the rhomb. The sizes of the rectangles were more than twice as large as those of their corresponding uncompensated rectangles and the size of the rectangle for the rhomb was much larger than that of the square.

## 4. Focusing and Wavefront Characteristics of a Spherical Mirror and HOEs

**Figure 6:**
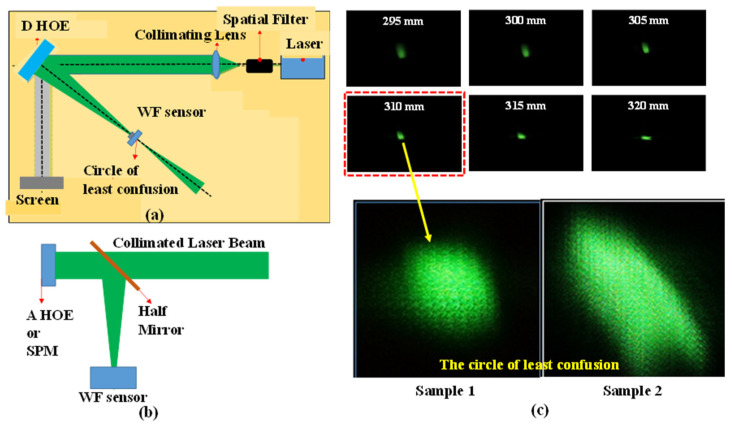
The two different optical setups used to measure the focal spot size and wavefront aberrations of the test subjects: (**a**) setup for the digitally recorded HOE, (**b**) setup for the analog HOE and SPM, and (**c**) the reflected beam shape changes close to the focusing position.

**Figure 7:**
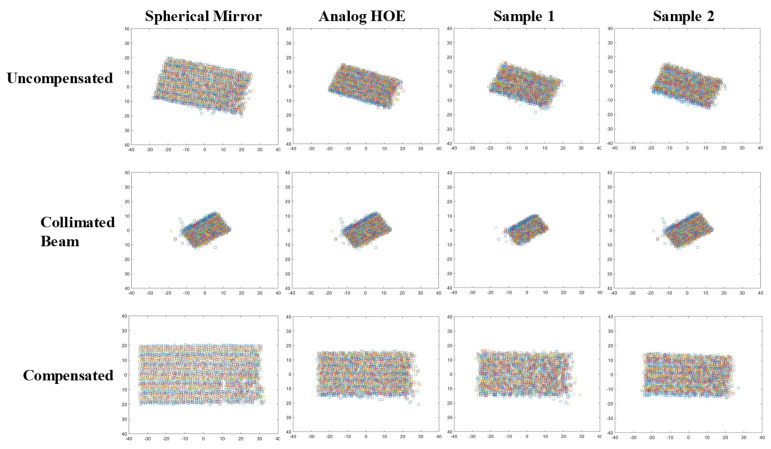
Wavefront aberrations of the samples, drawn by plotting the spot positions, compared with those of the spherical mirror and the analog HOE. The aberration distributions of the HOEs and the spherical mirror reveal rectangular shapes as the reconstructed images of the point hologram.

**Figure 8:**
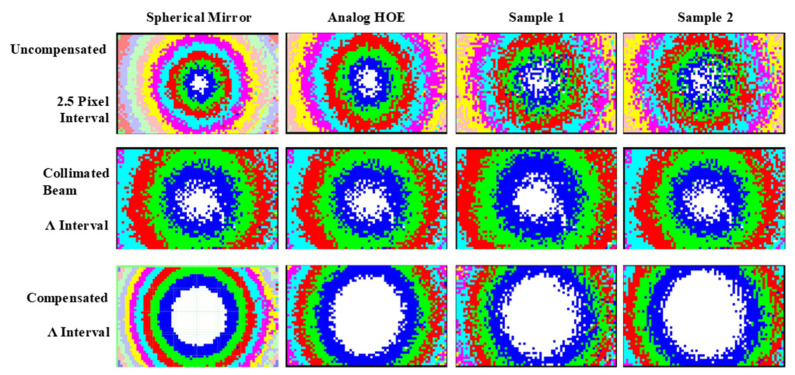
The phase maps of the four subjects. The phase maps in the top row were drawn using a 2.5-pixel interval, those in the second row corresponded to the collimated beams, and those in the bottom row corresponded to the compensated beams and were drawn using the wavelength interval.

For comparison with the optical characteristics of the digital HOE shown in Figure 2, a spherical mirror with a 220 mm focal length and an analog HOE for a heads-up display with a 500 mm focal length and a focal spot size of 5 mm were also prepared. Two different optical setups were used to measure the focal spot size and wavefronts of these test subjects, as shown in Figure 6. The collimated beam with a diameter of 50 mm was normally incident to the spherical mirror and the analog HOE, and the reflected beam was separated by a 45° half mirror on its path, as shown in Figure 6b. For the digital HOE samples, the collimated beam was incident to the center of each sample with a 38° beam incidence angle. In this case, a converging beam, which was diffracted from each sample, propagated normally to the sample with a 38° angle difference from the direction of the reconstruction beam. The samples were designed to have a focal length of 300 mm, but their circles of least confusion appeared at distances slightly greater than 300 mm. These were 310 mm and 315 mm for samples 1 and 2, respectively. The photos along the converging beam, which were taken at distances 295 mm to 320 mm from sample 1 with a 5 mm interval, showed an astigmatic focusing behavior because the beam was first focused at 300 mm but was elongated vertically and then focused again at 320 mm, but this time elongated horizontally. It measured approximately 2.2 × 2.2 mm and its shape was not a complete circle. The astigmatic distance was considered to be 20 mm, i.e., 300 mm to 320 mm. Sample 2 showed the same astigmatic behavior as sample 1, but the size of the circle of least confusion was 3.4 (horizontal) × 3.7 mm (vertical) and its shape resembled the broken half of a cucumber. It was more than twice as big as sample 1. In Figure 7, the wavefront deviation distributions of the samples are compared with those of the spherical mirror and the analog HOE. The deviation distributions of all the HOEs and the spherical mirror revealed rectangular shapes as the reconstructed images of the point hologram, as shown in Figure 5. However, they were almost centered at the (0.0) position, and the rotated angle and sizes of the rectangles differed from those of the holograms. The rectangles for the spherical mirror, analog HOE, and samples 1 and 2 of the digital HOE were rotated by −10°, −15°, −17°, and −17°, respectively. This indicated that from the spherical mirror to the samples the rotated angles were increasing but the sizes were decreasing. However, the angles and sizes of samples 1 and 2 were the same and had almost identical characteristics. Because the collimated beams for the spherical mirror, analog HOE, and samples 1 and 2 were constructed for each of them, the wavefronts of the collimated beams were measured individually. The wavefront deviation distribution of the collimated beams for the hologram, spherical mirror, analog HOE, and sample 2 were the same, as shown in Figure 5. However, the beam for sample 1 had the shape of a 27° rotated rectangle that was slightly smaller than those of the others, as shown in Figure 7. When they were compensated by the wavefront aberration of the collimated beam, the rectangles for the spherical mirror and the analog HOE became flat but samples 1 and 2 were still in the rotated form, although their rotation angle was around −2°. The rectangles became larger than the corresponding uncompensated rectangles, but their relative sizes had the same ratio as the uncompensated rectangles. The deviation distributions were extended from −34 to 32 (−20 to 20) pixels in the horizontal (vertical) direction, −27 to 27 (−16 to 16) pixels in the horizontal (vertical) direction, −26 to 24 (−14 to 15) pixels in the horizontal (vertical) direction, −27 to 23 (−15 to 14) pixels in the horizontal (vertical) direction for the spherical mirror, analog HOE, and samples 1 and 2, respectively. These extensions indicated that the convergence angles of the beams reflected from the spherical mirror and the analog HOE were higher than those of the beams diffracted from samples 1 and 2. This means that the focusability of the spherical mirror and the analog HOE was better than that of the samples. The wavefront characteristics of the four subjects were also compared using phase maps. Figure 8 shows the phase maps of the four subjects. Because each colored ring and the center circle represent the wavefronts that are to be converged at certain converging angles, the circle and rings will converge with smaller aberrations as their perfectness as rings and a circle increase. Therefore, the phase map provides a visualization of the presence of the wavefront aberration. In this regard, the compensated phase map for the spherical mirror shows near-ideal rings and circle shapes.

The phase map in the top row was drawn using a 2.5-pixel interval, and those for the collimated and compensated beams in the middle and bottom rows were drawn using the wavelength interval. These show that the right-hand edge of each map was noisier than the left side. This might be caused by some scratches on the right-hand edge of the microlens array. When the 50 mm collimated beam was incident to the spherical mirror with a 220 mm focal length, to the analog HOE with 500 mm focal length, and to the digital HOE samples with 300 mm focal lengths, the maximum converging angles of the reflected and diffracted beams should be ±6.48°, ±2.86°, and ±4.6°, respectively. However, the wavefront sensor could only detect a wavefront deviation up to −4.28° to 4.28° in the horizontal direction because of the 16:9 aspect ratio of the image detector in the mirrorless camera. When the diagonal directions were considered, the aberrations up to −6.04° to 6.04° can be detected. Therefore, the sensor was suitable for the HOEs but was insufficient for the spherical mirror. As shown in Figure 8, the uncompensated first row images show 12 rings, including one at the corners, for the spherical mirror, 8 rings for the analog HOE, and 7 rings for the two digital HOE samples in their horizontal direction. However, the rings for the two samples reveal barely identifiable boundaries compared with those of the spherical mirror and analog HOE. In the case of the compensated beams, nine very sharp concentric circular rings were identified for the spherical mirror, six rings for the analog HOE and sample 1, and five rings for sample 2. Because the last ring of each map was shown separately and the center of the rings was slightly shifted to the right-hand side, the actual numbers of rings should be 4.5 for the analog HOE, and 4 for samples 1 and 2. Therefore, the converging angles of the spherical mirror, analog HOE, sample 1, and sample 2 would be ±3.96°, ±2.80°, ±2.64°, and ±2.64°, respectively. The following observations were made regarding the maximum converging angles of the subjects: (1) the ±3.96° for the spherical mirror was much smaller than its maximum, because the 77 × 77 pixel array was insufficient to cover the convergence of the mirror; (2) the ±2.80° for the analog HOE closely matches its maximum; and (3) the ±2.64° for samples 1 and 2 was smaller than their maximum because their converging power was weaker. This is why the focused beam sizes of samples 1 and 2 were in the range of 5 to 9.6 mm^2^. Figure 8 also shows that the ring boundaries of the analog HOE were better defined and less noisy, i.e., a smaller number of different color points in each ring compared with the samples. The diameters of the white circles in the images increased in the order of spherical mirror, analog HOE, sample 1, and sample 2. These features indicated that the analog HOE converged more sharply than the samples even though its focal length was 1.7 (50/30) times longer than those of the samples, and the spherical mirror had a very sharply defined circular focus point. The radius of these rings was 11.9, 16.8, 20.5, 23.7, 26.6, 29.1, 31.4, 33.6, and 35.6 pixels, as shown in Table 1. From the sixth ring, the pixel increment corresponding to the 1 *λ* path length increment became less than 2.5 pixels. This number corresponded to the pixel number given in Figure 7.

## 5. Reconstructed Wavefronts of the Test Subjects

**Figure 9:**
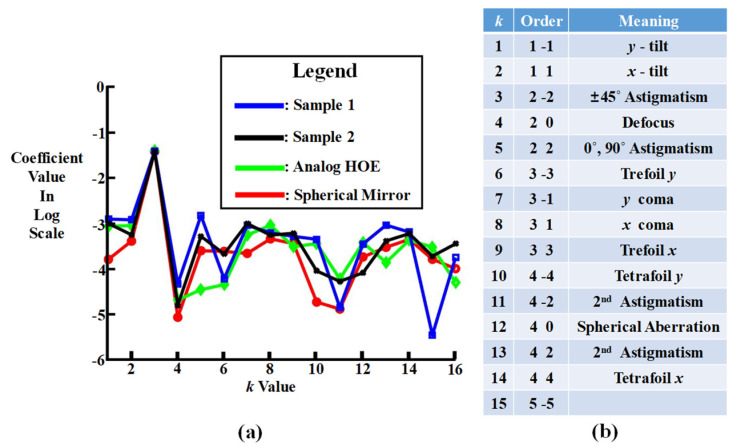
Fifteen coefficients of Zernike polynomials for the four test subjects, excluding the piston: (**a**) graphical presentation of the 15 coefficients and (**b**) the optical aberration represented by each coefficient.

**Figure 10:**
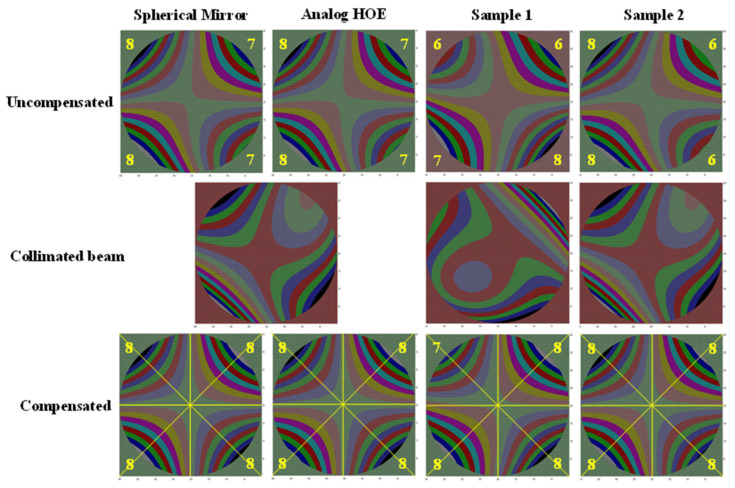
Reconstructed wavefronts of the four subjects, according to the Zernike coefficients in Figure 9. The compensated wavefronts look similar but those of the samples are less symmetric than those of the spherical mirror and analog HOE. The numbers in the four corners of each image represent the number of colored segments in each direction.

To reconstruct the wavefronts of the four test subjects from the wavefront aberration values, Zernike polynomials [13] were used. The wavefront $z\left(x,y\right)$ is expressed by the polynomials as:

(1) $$z\left(x,y\right)=\sum_{k}^{n}Z_{k}\varphi_{k}\left(x,y\right),$$

where $Z_{k}$ and $\varphi_{k}\left(x,y\right)$ are the *k*^th^ coefficient and component of the Zernike polynomials, respectively. The coefficient represents the root mean square error of the optical aberration represented by its corresponding component. Because Equation (1) can be represented by a two-dimensional matrix of *x* and *y*, $Z_{k}$ is calculated using the least square method [18] as:

(2) $$C={\left(A^{T}A\right)}^{-1}\left(AB\right),$$

where C is the 15 element matrix of $Z_{k},$ *A* is a two-dimensional matrix form of $\varphi_{k}\left(x,y\right)$, $A^{T}$ is a transpose matrix of *A*, and *B* is a two-dimensional matrix form of $z\left(x,y\right)$. Figure 9 compares 15 coefficients of the Zernike polynomials, i.e., *n* = 15 for the four test subjects, excluding the piston, i.e., the constant term. It also shows the optical aberration represented by each coefficient. In Figure 9a, the *y*-axis of the graph is in log scale and the *x*-axis represents the *k* value. It shows that the spherical mirror had the smallest values and was followed by the analog HOE, sample 1, and sample 2 for most coefficient values, even though they were mostly in the range of 10^−3^. There was little difference in the coefficient values for the subjects. The highest coefficient value was k=3, i.e., ±45° astigmatism term. The wavefronts of the subjects reconstructed from the coefficients are shown in Figure 10. Because the polynomials are defined within a unit circle, in each image the reconstructed wavefront of the subject is drawn within the circle. The wavefront aberrations of the subjects were within the wavelength range of −0.04 *λ*~0.04 *λ*. The 45° diagonal represents 0.0 to −0.04 *λ* and 135° 0.0 to 0.04 *λ*. The colored segments in each diagonal represent a ±0.005 *λ* interval. Consequently, there can be eight colored segments for each direction of each wavefront. The color in the center of the circle and outside it represents 0.0 *λ*. The wavefront aberration increases further from the center of the circle. The number of colored segments specified in each corner of each square reveals the symmetricity of the wavefront. As indicated by the numbers in each wavefront, there were eight colored segments for the compensated wavefronts of the subjects, with the exception of sample 1. Sample 1 revealed seven colored segments on its upper left-hand side. According to the size and shape of the colored segments in the circumference, the wavefront symmetricity decreases in the order of the spherical mirror, analog HOE, sample 2, and sample 1. However, the overall symmetricity of the wavefront decreases in the order of the spherical mirror, analog HOE, and samples 1 and 2, as indicated by the two diagonal lines and the horizontal and vertical bisect lines. The pin-cushion pattern in the middle of each wavefront reveals the decrease. The symmetricities of the opposite-facing patterns along the lines deteriorate further between the spherical mirror to sample 2. This means that the reconstructed wavefronts of samples 1 and 2 were more distorted. It is also noticeable that the uncompensated wavefronts were influenced by their corresponding collimated beams. This is clearly demonstrated by the wavefronts of samples 1 and 2. The wavefronts of the uncompensated and collimated beams for sample 1 were simply the sample 2 wavefronts rotated 180°. The compensated wavefronts did not reveal this property.

## 6. Conclusions

The digital HOE can be used as an optical component, but its optical characteristics are somewhat inferior compared with the spherical mirror and the analog HOE because it reveals a more distorted reconstructed wavefront and a noisier phase map than the spherical mirror and the analog HOE. It also shows astigmatism as the reconstructed image of a hologram displayed on an SLM. To make a digital HOE that has the same characteristics as the analog HOE, the number of defective hologram points need to be minimized and the astigmatism eliminated. In addition, the time to manufacture the HOE should also be minimized as it is currently too long. It was also observed that the phase map could visualize the presence and possible quantity of wavefront aberrations in HOEs and optical components in the same way as the reconstructed wavefront from the Zernike polynomials. The phase map could represent a convenient tool for estimating the optical performance of all the optical components.

## Figures and Tables

**Table 1:** Wavefront aberration angles corresponding to nλ (a) and 2.5 m (b).

**(a)**		
**Wavelength Differences**	**Deviation Angle**	**Pixel Numbers**
1𝜆	$1.3214^{\circ }$	11.9
2λ	$1.8685^{\circ }$	16.8
3𝜆	$1.8685^{\circ }$	20.5
4λ	$2.6419^{\circ }$	23.7
5𝜆	$2.9534^{\circ }$	26.5
6λ	$3.2349^{\circ }$	29.1
7𝜆	$3.4937^{\circ }$	31.4
8λ	$3.7346^{\circ }$	33.6
9𝜆	$3.9607^{\circ }$	35.6
10λ	$4.1744^{\circ }$	37.5
11λ	$4.3777^{\circ }$	39.4
**(b)**		
**Pixel Numbers**	**Deviation Angle**	
2.5	$0.2786^{\circ }$	
5.0	$0.5572^{\circ }$	
7.5	$0.8357^{\circ }$	
10.0	$1.1143^{\circ }$	
12.5	$1.3927^{\circ }$	
15.0	$1.6711^{\circ }$	
17.5	$1.9495^{\circ }$	
20.0	$2.2277^{\circ }$	
22.5	$2.5058^{\circ }$	
25.0	$2.7838^{\circ }$	
27.5	$3.0617^{\circ }$	
30.0	$3.3394^{\circ }$	
32.5	$3.6170^{\circ }$	
35.0	$3.8944^{\circ }$	
37.5	$4.1716^{\circ }$	
40.0	$4.4487^{\circ }$	

## Data Availability

The data in this article were solely prepared for this article.
